# C-type lectin-like domain family 2 (CLEC2D) promotes proliferation and migration of breast cancer and serves as a poor prognostic factor

**DOI:** 10.1007/s12282-025-01777-5

**Published:** 2025-09-12

**Authors:** Mio Yamaguchi-Tanaka, Yui Kurihara, Kiyoshi Takagi, Ai Sato, Iori Yasuda, Yuto Yamazaki, Minoru Miyashita, Takashi Suzuki

**Affiliations:** 1 Department of Pathology and Histotechnology, Tohoku University Graduate School of Medicine, 2-1 Seiryo-Machi, Aoba-Ku, Sendai, Miyagi-Ken 980-8575 Japan; 2 Department of Pathology, Tohoku University Hospital, 1-1 Seiryo-Machi, Aoba-Ku, Sendai, Miyagi 980-8574 Japan; 3 Department of Breast and Endocrine Surgical Oncology, Tohoku University Graduate School of Medicine, 2-1 Seiryo-Machi, Aoba-Ku, Sendai, Miyagi 980-8575 Japan; 4 Department of Anatomic Pathology, Tohoku University Graduate School of Medicine, 2-1 Seiryo-Machi, Aoba-Ku, Sendai, Miyagi 980-8575 Japan

**Keywords:** Breast cancer, C-type lectin-like domain family 2 (CLEC2D), Tumor microenvironment, Immunohistochemistry, Prognostic factor

## Abstract

**Background:**

C-type lectin-like domain family 2 (CLEC2D), a transmembrane protein, is a ligand for the inhibitory receptor CD161, which is expressed in several types of immune cells. CLEC2D expressed on cancer cells suppresses antitumor effect of these cells by interacting with CD161 in human malignancies. However, its clinical significance in breast cancer and its direct biological role in cancer cells remain largely unclear.

**Methods:**

In this study, we immunolocalized CLEC2D in 174 breast cancer tissues and correlated its immunoreactivity with clinicopathological parameters and clinical outcomes. Additionally, we conducted in vitro assays to examine the effects of CLEC2D on the proliferation and migration of breast cancer cell lines.

**Results:**

CLEC2D immunoreactivity was predominantly detected in the cytoplasm of breast cancer cells and was associated with increased proliferation and invasion, as well as poor clinical outcomes especially in those who had received chemotherapy. In vitro experiments demonstrated that the knockdown of CLEC2D significantly suppressed the proliferation and migration of MCF-7, MDA-MB-231, T-47D breast cancer cells.

**Conclusion:**

We therefore concluded that CLED2D directly promoted breast cancer cell proliferation and migration independently of immune cells and served as a poor prognostic factor in breast cancer.

**Supplementary Information:**

The online version contains supplementary material available at 10.1007/s12282-025-01777-5.

## Introduction

Breast cancer is globally the most diagnosed malignant tumor in women. Recent research has revealed its biological characteristics, leading to widespread use of endocrine therapies targeting estrogen signaling and cytotoxic chemotherapy, both of which have significantly improved patient outcomes. However, many patients develop de novo or acquired resistance, particularly to chemotherapy, highlighting the urgent need to identify new therapeutic targets to further improve clinical outcomes [1].

C-type lectin-like domain family 2 (CLEC2D), also known as lectin-like transcript 1 (LLT1) and osteoclast inhibitory lectin, is a transmembrane protein belonging to the C-type lectin-like protein family [2]. CLEC2D is a ligand for the inhibitory receptor CD161 on several types of immune cells such as natural killer (NK) cells, CD4 + T cells, CD8 + T cells, mucosal associated invariant T (MAIT) cells, and natural killer T (NKT) cells [3, 4] to transduce inhibitory signals to suppress cytotoxic effect of these cells. NK cells play a crucial role in the innate immune system, particularly in identifying and eliminating cancer cells and infected cells. The cytotoxicity of NK cells is regulated by a balance of activating and inhibitory signals mediated by their receptors and ligands on target cells [5]. However, during carcinogenesis, abnormal cells often overexpress inhibitory ligands such as CLEC2D to suppress NK cell function, which in turn establishes the escape from NK cells [6]. Previous in vitro analysis has shown that CLEC2D is highly expressed in cancer cells and suppresses the cytotoxicity and cytokine production of NK cells by interacting with CD161 [7, 8]. CD8⁺ T cells are key antitumor effectors, but exhausted CD8⁺ T cells (CD8Tex), in which CLEC2D has recently been identified as a hub gene in human breast cancer, are common in tumors [9]. Alvarez Calderon et al. reported high CLEC2D expression in hematologic malignancies and showed that blocking CD161 enhanced T-cell cytotoxicity and proliferation [10].

CLEC2D expression has been immunohistochemically examined in several human malignancies including glioma, prostate cancer, head and neck cutaneous squamous cell carcinoma (HNcSCC), clear cell renal cell carcinoma (ccRCC), and oral squamous cell carcinoma, where CLEC2D is often overexpressed and identified as a poor prognostic factor [11–13]. However, its clinical significance in breast cancer remains largely unclear. Additionally, infiltration of NK cells is rarely observed in the tumor microenvironment due to already established immune escape mechanisms from NK cells [14]. Therefore, the biological role of CLEC2D is still to be clarified in breast cancer.

Here, we immunolocalized CLEC2D in 174 human breast carcinoma tissues and evaluated its clinical significance in breast cancer. Additionally, we conducted in vitro studies to further investigate the NK cell-independent role of CLEC2D in breast cancer.

## Materials and methods

### Patients and tissues

A total of 174 invasive breast cancer specimens were collected from patients who underwent surgical resection at Tohoku University Hospital between 2006 and 2008. All samples were fixed in 10% neutral buffered formalin and embedded in paraffin. Clinical outcomes were assessed based on disease-free survival (time from surgery to locoregional or distant metastasis) and breast cancer-specific survival (time from surgery to breast cancer-related death). The median follow-up periods were 63 months (range, 3–108) and 64 months (range, 3–108), respectively. Ninety-one patients had received neoadjuvant, adjuvant chemotherapy, or both. One case lacked chemotherapy information and was excluded from survival analysis stratified by chemotherapy status. This study was approved by the Ethics Committee of the Tohoku University School of Medicine.

### Immunohistochemistry

Anti-CLEC2D antibody was obtained from Abnova (clone 4C7; Taipei, Taiwan). Antigen retrieval was performed using an autoclave in Histofine pH 9 buffer (Nichirei Biosciences, Japan) at 121 °C for 10 min. The antigen–antibody complex was detected using the Histofine Kit (Nichirei Biosciences) and 3, 3′-diaminobenzidine, followed by hematoxylin counterstaining as described previously [15]. Human placenta was used as positive control. The cases with immunoreactivity in more than 10% of carcinoma cells were classified as CLEC2D-positive, based on the criteria previously used to evaluate immunostaining [16]. Immunohistochemical status of estrogen receptor (ER), progesterone receptor (PR), human epidermal growth factor receptor 2 (HER2), and the Ki67 labeling index (LI) were based on previous studies [17]. Normal mammary epithelium was evaluated within invasive breast cancer specimens.

### Cell lines and chemicals

Human breast cancer cell lines MCF-7 and MDA-MB-231 were obtained from the American Type Culture Collection (Manassas, VA, USA). T-47D was sourced from the Research Bioresources Cell Bank (Osaka, Japan). All cell lines were cultured in RPMI-1640 medium (Fujifilm Wako, Osaka, Japan) supplemented with 10% fetal bovine serum (Gibco, Rockville, MD, USA) and maintained at 37 °C in a 5% CO₂ atmosphere. Epirubicin (EPI) and docetaxel (DTX) were purchased from Fujifilm Wako.

### Small interfering RNA transfection

Two siRNAs targeting CLEC2D (5′-GAGGUUUUGUGACUCACAAdTdT-3′ and 5′-UCAUGUUUCUGACAAUCAUdTdT-3′) were obtained from Ajinomoto Bio-Pharma Services, Inc. (Osaka, Japan). Two siRNAs targeting CLEC2D were mixed at a 1:1 ratio and used as a siRNA cocktail for CLEC2D (siCLEC2D cocktail) to reduce off-target effects of RNA interference. The MISSION siRNA Universal Negative Control (Sigma-Aldrich, St. Louis, MO, USA) was used as a negative control (siCTRL). siRNA transfection (MCF-7; 10 nM, MDA-MB-231 and T-47D; 20 nM) was performed using the Lipofectamine RNAi MAX transfection reagent (Thermo Fisher Scientific, Waltham, MA, USA) following the manufacturer’s protocol.

### Real-time PCR

RNA extraction and cDNA synthesis were performed as per the methodology described in previous reports [18]. Real-time PCR was conducted using the THUNDERBIRD SYBR qPCR Mix (TOYOBO) on a LightCycler Nano system (Roche Diagnostics Japan). Relative CLEC2D mRNA levels were normalized to glyceraldehyde-3-phosphate dehydrogenase (GAPDH) expression. The primer sequences for CLEC2D and GAPDH were as follows; CLEC2D, 5′-TGCCCAGAAAGCTGGATTGG-3′ (forward) and 5′-AACCTGAGCAAGATCAGCATC-3′ (reverse) and GAPDH, 5′-GCCTTCTCCATGGTGGTGAA-3′ (forward) and 5′-CCATCTTCCAGGAGCGAGATC-3′ (reverse).

### Cell proliferation assay

Breast cancer cells were transfected with the siCLEC2D cocktail in a 96-well culture plate. Cell proliferation was assessed over four days by the WST-8 method using the Cell Counting Kit-8 (Dojindo Molecular Technologies, Inc., Kumamoto, Japan). Absorbance at 450 nm was measured using a Bio-Rad iMark plate reader (Bio-Rad Laboratories Inc., Hercules, CA, USA).

### Cell migration assay

The migration of breast cancer cells was evaluated using a wound healing assay with culture inserts (Platypus Technologies, Madison, WI, USA). Cells transfected with the siCLEC2D cocktail were seeded into a 96-well plate with culture inserts at > 90% confluency. After the inserts were removed, the gaps were analyzed using ImageJ 1.52a software (https://imagej.nih.gov/ij/, accessed on 19 January 2025). The migration ability was evaluated as the percentage of the gap area relative to its size at the time of culture insert removal (0 h).

### Statistical analysis

Statistical analysis for immunohistochemistry was conducted using JMP Pro 17.0.0 software (SAS Institute, Cary, NC, USA). The correlation between clinicopathological parameters and CLEC2D immunoreactivity was assessed using the χ^2^ test or the Mann–Whitney U test. Survival curves were generated using the Kaplan–Meier method and evaluated with the log rank test. Both univariate and multivariate analyses were performed using the Cox proportional hazards model. In vitro experiments were analyzed using Scheffé’s F test with StatView 5.0 J software (SAS Institute) and Dunnett's test with JMP Pro 17.0.0 software. Data are presented as the mean ± standard deviation (SD). P-value of < 0.05 was considered statistically significant.

## Results

### Immunohistochemistry for CLEC2D in human breast carcinoma tissues

**Fig. 1:**
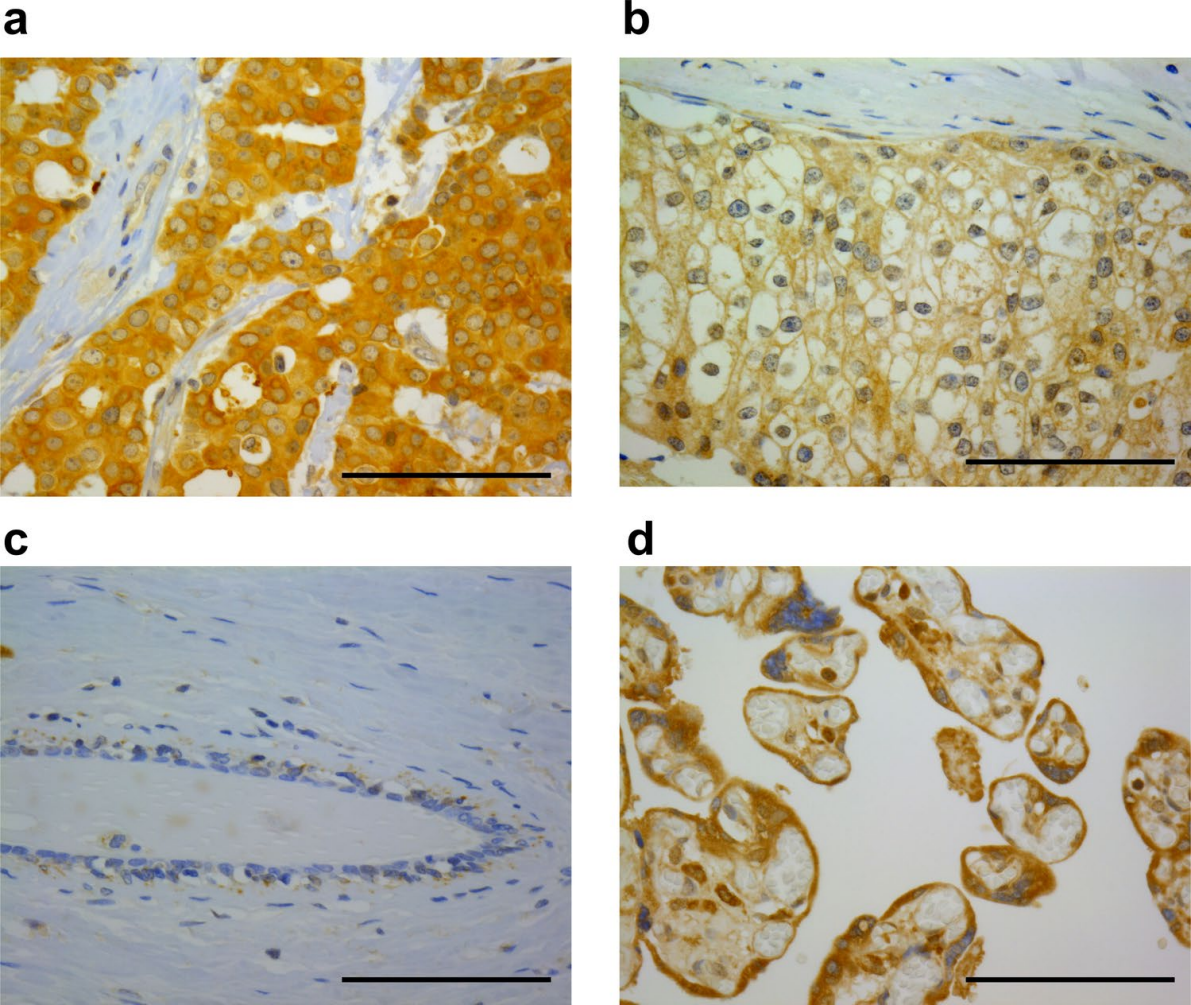
Immunoreactivity of CLEC2D in human breast carcinoma tissues. **a–c** CLEC2D immunoreactivity (brown) was observed in both the cytoplasm (**a**) and membrane (**b**) of breast carcinoma cells, with no staining detected in the surrounding connective tissue or normal breast epithelium (**c**). **d** The placenta was used as a positive control, showing CLEC2D immunoreactivity in syncytiotrophoblasts and cytotrophoblast. Bar = 100 μm, respectively

CLEC2D immunoreactivity was observed in both the cytoplasm (Fig. 1a) and cell membrane of breast carcinoma cells (Fig. 1b), while it was negligible in normal breast epithelium (Fig. 1c). In the positive control (placenta), CLEC2D immunoreactivity was detected in the cytoplasm of syncytiotrophoblasts and cytotrophoblasts (Fig. 1d). A total of 79 patients (45%) were classified as positive for CLEC2D. The association between clinicopathological parameters and CLEC2D immunoreactivity is summarized in Table 1. CLEC2D immunoreactivity was positively correlated with the pathological T factor (P = 0.030), Ki67 LI (P = 0.025), and receipt of neoadjuvant chemotherapy (P = 0.0026), while it showed an inverse correlation with PR status (P = 0.021).

**Table 1:** Clinicopathological characteristics of CLEC2D in breast carcinoma tissues (n = 174)

	CLEC2D		
	Negative (n = 95)	Positive (n = 79)	P
Age^*^	55 (27–87)	56 (32–85)	0.62
Menopausal status			
Pre-	40	26	0.21
Post-	55	53	
pT			
pT1	69	45	**0.030**
pT2–4	26	34	
Lymph node metastasis			
Negative	70	50	0.14
Positive	25	29	
Stage			
1	60	38	0.13
2	22	24	
3	13	17	
Histological grade			
1	37	24	0.37
2	38	39	
3	20	14	
ER			
Negative	14	18	0.17
Positive	81	61	
PR			
Negative	24	33	**0.021**
Positive	71	46	
HER2			
Negative	83	65	0.35
Positive	12	14	
Ki67 LI^*^	9 (1–72)	16 (1–60)	**0.025**
Neoadjuvant chemotherapy			
Treated	12	25	**0.0026**
Untreated	82	54	

^*^Data was presented as median (minimum–max)

All other values represent the number of cases. P < 0.05 was considered significant and described as bold

### Association between immunoreactivity of CLEC2D and clinical outcome in breast cancer patients

**Fig. 2:**
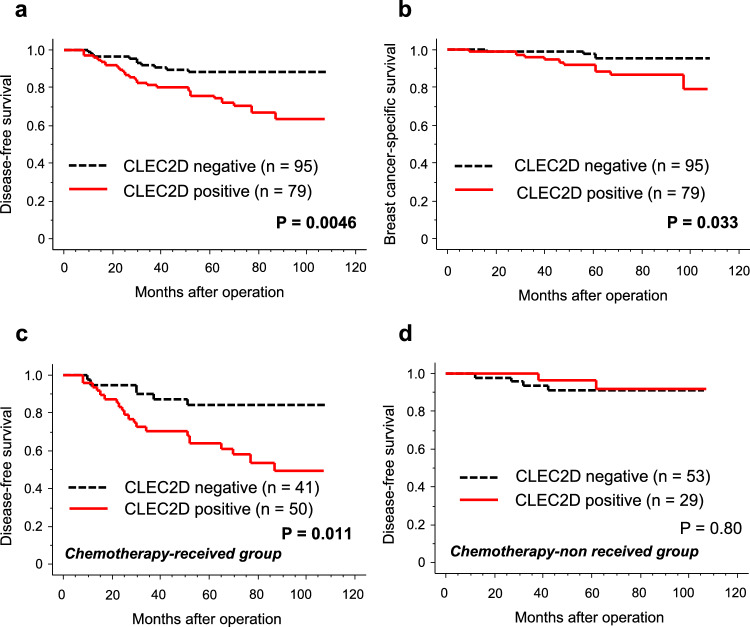
Prognostic analysis according to CLEC2D immunoreactivity. Disease-free survival curves (**a**) and breast cancer-specific survival curves (**b**) according to CLEC2D immunoreactivity in breast carcinoma cells (n = 174), and those who had received (**c**, n = 91) or not received chemotherapy (**d**, n = 82). Survival curves were generated by Kaplan–Meier method and statistical analysis was performed using log lank test.

We subsequently examined the relationship between CLEC2D immunoreactivity and clinical outcomes, as assessed by disease-free survival and breast cancer-specific survival. CLEC2D immunoreactivity was significantly correlated with shorter disease-free (Fig. 2a,   P = 0.0046) and breast cancer-specific survival (Fig. 2b,   P = 0.033). The results of the univariate and multivariate analyses were summarized in Table 2. Univariate analysis identified CLEC2D as well as pT, lymph node metastasis, histological grade, ER, PR, and Ki67 LI as significant prognostic factors for disease-free survival, while subsequent multivariate analysis revealed that pT (P = 0.012) and Ki67 LI (P = 0.0065) were independent prognostic factors for disease-free survival. Similarly, CLEC2D as well as pT, lymph node metastasis, histological grade, PR, and Ki67 LI were turned out to be significant prognostic factors for breast cancer specific survival, while none of them were independent prognostic factors.

**Table 2:** Uni- and multivariate analysis of disease-free and breast cancer-specific survival in 174 breast cancer patients

	Disease-free survival				Breast cancer-specific survival			
	Univariate		Multivariate		Univariate		Multivariate	
	P	relative risk (95% CI)	P	relative risk (95% CI)	P	relative risk (95% CI)	P	relative risk (95% CI)
pT (pT2–4/pT1)	** < 0.0001**	**4.7** **(2.3–9.6)**	**0.012**	**3.2** **(1.3–8.1)**	**0.0014**	**11.7** **(2.6–53)**	0.059	5.2 (0.94–29)
Lymph node metastasis (Positive/Negative)	**0.0043**	**2.7** **(1.4–5.4)**	0.91	1.0 (0.45–2.4)	**0.0045**	**5.5** **(1.7–18)**	0.42	1.7 (0.45–6.8)
Histological grade (3/1 + 2)	**0.013**	**2.5** **(1.2–5.1)**	0.30	0.58 (0.21–1.6)	**0.018**	**3.9** **(1.3–12)**	0.51	1.8 (0.31–10)
ER (Negative/Positive)	**0.0053**	**2.8** **(1.4–5.6)**	0.81	1.1 (0.39–3.3)	0.080	2.7 (0.89–8.3)		
PR (Negative/Positive)	**0.0009**	**3.3** **(1.6–6.5)**	0.073	2.3 (0.92–5.8)	**0.016**	**4.3** **(1.3–14)**	0.10	3.3 (0.79–14)
HER2 (Negative/Positive)	0.11	3.2 (0.76–13)			0.39	2.4 (0.32–19)		
Ki67 LI (≥ 20%/< 20%)	** < 0.0001**	**4.8** **(2.4–9.7)**	**0.0065**	**3.5** **(1.4–8.6)**	**0.0081**	**4.6** **(1.5–14)**	0.84	1.2 (0.20–7.3)
CLEC2D (Positive/Negative)	**0.0067**	**2.8** **(1.3–5.9)**	0.11	1.9 (0.87–4.1)	**0.047**	**3.7** **(1.0–14)**	0.23	2.3 (0.59–8.8)

*CI* 95% confidence interval

P < 0.05 (bold) was considered significant, and incorporated in the multivariate analysis

When we correlated CLEC2D with clinical outcomes according to the status of chemotherapy, CLEC2D immunoreactivity was significantly correlated with shorter disease-free survival in the patients who had received chemotherapy (Fig. 2c,  P = 0.011), while no significant correlation was observed in those without chemotherapy (Fig. 2d,  P = 0.80). Similarly, univariate analysis also demonstrated that CLEC2D immunoreactivity was associated with increased risk of recurrence in chemotherapy-received group as demonstrated by univariate analysis, multivariate analysis failed to detect statistical significance between them (Table 3, P = 0.058). PR (P = 0.0099), HER2 (P = 0.0051), and Ki67 LI (P = 0.015) were independent prognostic factors for disease-free survival in breast cancer patients who had received chemotherapy.

**Table 3:** Uni- and multivariate analysis of disease-free and breast cancer-specific survival in 91 breast cancer patients who had received chemotherapy

	Disease-free survival			
	Univariate		Multivariate	
	P	relative risk (95% CI)	P	relative risk (95% CI)
pT (pT2–4/pT1)	**0.011**	**3.1 (1.3–7.3)**	0.072	2.3 (0.93–5.7)
Lymph node metastasis (Positive/negative)	0.30	1.5 (0.69–3.3)		
Histological grade (3/1 + 2)	0.57	1.3 (0.57–2.7)		
ER (Negative/positive)	0.16	1.7 (0.81–3.8)		
PR (Negative/positive)	**0.046**	**2.3 (1.0–5.3)**	**0.0099**	**3.2 (1.3–7.6)**
HER2 (Negative/positive)	**0.041**	**4.48 (1.1–19)**	**0.0051**	**8.4 (1.9–38)**
Ki67 LI (≥ 20%/< 20%)	**0.026**	**2.4 (1.1–5.2)**	**0.015**	**2.8 (1.2–6.6)**
CLEC2D (Positive/negative)	**0.016**	**3.1 (1.2–7.6)**	0.058	2.5 (0.97–6.5)

*CI* 95% confidence

P < 0.05 (bold) was considered significant, and incorporated in the multivariate analysis

### Effects of CLEC2D on cell proliferation and migration in breast cancer cells

**Fig. 3:**
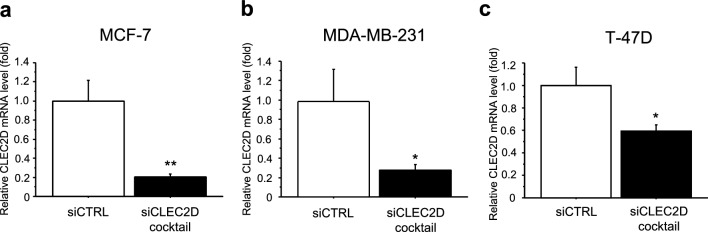
The effects of CLEC2D knockdown in breast cancer cell lines. CLEC2D mRNA levels in MCF-7 (**a**), MDA-MB-231 (**b**), and T-47D cells (**c**) transfected with CLEC2D-specific siRNA cocktail. * P < 0.05, *** P < 0.001 vs. control (siCTRL). The data are presented as the mean ± S.D. (n = 3). Statistical analysis was performed using Scheffé’s F test. mRNA levels were normalized to the expression of GAPDH.

**Fig. 4:**
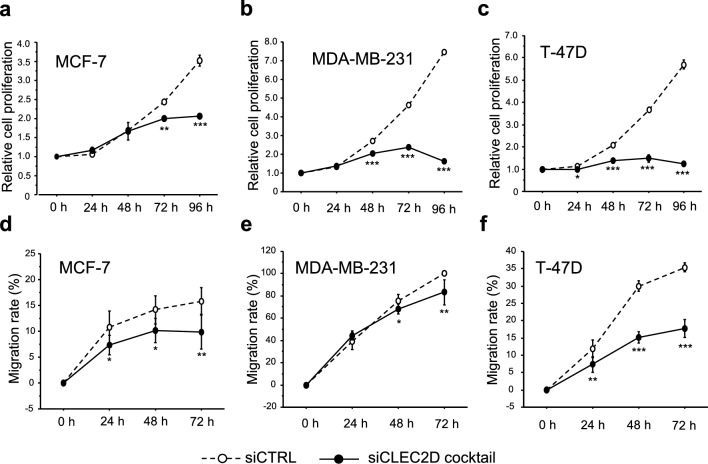
The effects of CLEC2D knockdown in breast cancer cell lines on cell proliferation and migration. Cell proliferation assay (**a–c**) and migration assay (**d–f**) in MCF-7 (**a, d**), MDA-MB-231 (**b, e**), and T-47D cells (**c, f**) transfected with CLEC2D-specific siRNA cocktail. * P < 0.05, ** P < 0.01, *** P < 0.001 vs. control (siCTRL). Statistical analysis was performed using Scheffé’s F test. The data are presented as the mean ± S.D. (n = 3 for cell proliferation assay and n = 6 for migration assay)

We investigated the effects of CLEC2D on the proliferation and migration of breast cancer cells using three breast cancer cell lines. CLEC2D-specific siRNA cocktail was transfected into MCF-7 (Fig. 3a), MDA-MB-231 (Fig. 3b), and T-47D (Fig. 3c), and decreased CLEC2D mRNA expression was confirmed by real-time PCR. Cell proliferation activity was significantly reduced upon transfection with the siCLEC2D cocktail compared to the siCTRL in all cell lines: MCF-7 (Fig. 4a), MDA-MB-231 (Fig. 4b), T-47D (Fig. 4c). Similarly, CLEC2D knockdown suppressed the migration ability of MCF-7 (Fig. 4d), MDA-MB-231 (Fig. 4e), T-47D (Fig. 4f). Furthermore, we evaluated cell invasion ability using a Boyden chamber assay with Matrigel-coated transwells. Contrary to expectations, cell invasion was significantly increased in MCF-7 cells transfected with the siCLEC2D cocktail (Supplementary Fig. 1a), whereas it was reduced in MDA-MB-231 cells (Supplementary Fig. 1b). T47D cells exhibited intrinsically low invasive potential and invasion was scarcely observed regardless of siCLEC2D transfection (data not shown).

We further analyzed the mRNA expression of CLEC2D-related molecules, CD161 (the receptor for CLEC2D), as well as toll-like receptors TLR2 and TLR4, noting that CLEC2D has been reported to form heterodimers specifically with TLR2 [19]. CD161 mRNA was almost undetectable in all three breast cancer cell lines (data not shown). TLR2 mRNA was detected in both MCF-7 and MDA-MB-231 cells, and was suppressed in MCF-7 cells transfected with siCLEC2D (Supplementary Fig. 2a). Although TLR4 mRNA was detected in MDA-MB-231 cells, CLEC2D silencing did not affect TLR4 expression (Supplementary Fig. 2b). TLR2 mRNA in T-47D cells and TLR4 mRNA in MCF-7 and T-47D cells were at the lower limit of detection.

### Regulation of CLEC2D expression in breast cancer cells following chemotherapy

**Fig. 5:**
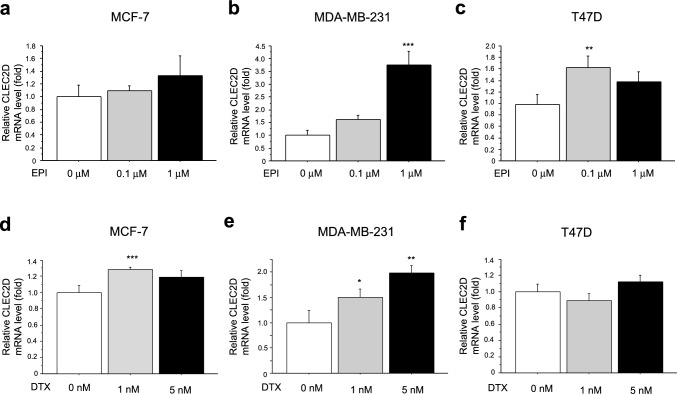
The expression of CLEC2D mRNA in breast cancer cell lines after chemotherapy. CLEC2D mRNA levels in MCF-7 (**a, d**), MDA-MB-231 (**b, e**), and T-47D (**c, f**) cells treated with epirubicin (EPI, **a–c**) and docetaxel (DTX, **d–f**) for 48 h were evaluated by real time PCR. * P < 0.05, ** P < 0.01, *** P < 0.001 vs. control (0 μM). Statistical analysis was performed using Dunnett's test. The data are presented as the mean ± S.D. (n = 3). mRNA levels were normalized to the expression of GAPDH.

We then evaluated CLEC2D mRNA in MCF-7 (Fig. 5a, d), MDA-MB-231 (Fig. 5b,e), and T-47D (Fig. 5c,f) cells treated with EPI (a–c) and docetaxel (DTX, d–f) for 48 h. CLEC2D expression was upregulated in MDA-MB-231 and T-47D cells treated with EPI, and MCF-7 and MDA-MB-231 cells treated with DTX. We further validated these findings using EPI-resistant breast cancer cell lines (Supplementary Fig. 3a) along with their parental chemo-sensitive counterparts. CLEC2D expression was significantly increased in both MCF-7-EPI-R and MDA-MB-231-EPI-R cells. In addition, we analyzed the mRNA expression of TLR2 and TLR4 in both MCF-7-EPI-R and MDA-MB-231-EPI-R cells. While TLR2 mRNA was upregulated in MCF-7-EPI-R cells, both TLR2 and TLR4 mRNA were suppressed in MDA-MB-231-EPI-R cells (Supplementary Fig. 3b, c).

## Discussion

In the present study, we first investigated the clinicopathological significance of CLEC2D in human breast carcinoma tissues by immunohistochemistry. We then investigated the effects of CLEC2D using three well-characterized breast cancer cell lines [20]. We used both ER-negative (MDA-MB-231) and ER-positive (MCF-7 and T-47D) cell lines because immunohistochemistry showed no correlation between CLEC2D expression and ER status. Immunohistochemistry also suggested that CLEC2D is associated with chemotherapy resistance. Since MDA-MB-231 and T-47D harbor p53 mutations while MCF-7 retains wild-type p53, their responses to chemotherapy-induced apoptosis differ, therefore, we selected these cell lines. In immunohistochemical analysis, CLEC2D immunoreactivity was predominantly detected in the breast cancer cells compared to normal breast epithelium, suggesting the possibility that CLEC2D has a pivotal role in breast cancer. CLEC2D was expressed on both the cytoplasm and cell membrane of breast cancer cells. While CLEC2D is expressed on the cell membrane to interact with CD161, cytoplasmic—especially in endosomes—expression of CLEC2D in macrophages has also been reported. CLEC2D recognizes damage-associated molecular patterns (DAMPs) and transports them into the cytoplasm via endocytosis, where it regulates NF-κB signaling and cytokine expression [21, 22]. Furthermore, CLEC2D was localized in the cytoplasm of oropharyngeal squamous cell carcinomas and ccRCC carcinomas [12, 13]. Taken together, CLEC2D in the cell membrane likely plays an important role in ligand binding, while internalized CLEC2D in the cytoplasm may also contribute to cytokine expression and activation of signaling pathways, potentially affecting tumor progression.

CLEC2D expression was positively correlated with poorer clinical outcomes, including shorter disease-free and breast cancer-specific survival, consistent with findings in other malignancies [11–13]. Its stronger impact on disease-free survival likely reflects differences in event numbers (recurrence: 33 cases; breast cancer-related death: 13 cases). Multivariate analysis showed CLEC2D was not an independent prognostic factor, whereas pT and Ki67 were, possibly reflecting CLEC2D’s role in promoting proliferation and migration. In addition, the favorable response to combination therapy with anti-HER2 agents and chemotherapy contributes to HER2 being considered a positive prognostic factor.

It was of importance that the correlation between CLEC2D expression and post-operative recurrence was predominantly observed in chemotherapy-received group. Furthermore, CLEC2D was frequently expressed in breast cancer with neoadjuvant chemotherapy, and its expression was upregulated by EPI in MDA-MB-231 and T-47D cells, and by DTX in MCF-7 and MDA-MB-231 cells. CLEC2D is induced by TLR3/4/7/8/9 ligands in immune cells [23], and these TLRs are also expressed in breast cancer cells [24]. The TLR family acts as receptors for DAMPs, which are upregulated in response to chemotherapy-induced DNA damage [25], suggesting that CLEC2D is upregulated via TLR activation by DAMPs following chemotherapy, contributing to chemoresistance. However, in this study, CLEC2D expression was elevated in epirubicin-resistant MDA-MB-231 cells, while TLR4 expression was markedly reduced, suggesting that TLR4 may not play a significant role in regulating CLEC2D in these cells. In addition, although no significant changes in CLEC2D expression were observed in MCF-7 cells exposed to EPI or in T-47D cells exposed to DTX, the profiles of DAMPs secreted in response to various anticancer drugs and the expression of their receptors, TLRs, likely vary depending on the breast cancer cell line [24, 26]. This complexity in ligand–receptor expression may explain why not all cell lines exhibit the same changes. In addition, CLEC2D is elevated in immune cells of acute lymphoblastic leukemia but decreased in T cells after chemotherapy [27]. While our study found increased CLEC2D in breast cancer cells after chemotherapy, this response may vary among cell types. CD161, expressed in CD8⁺ T cells, interacts with CLEC2D to impair cytotoxic T-cell antitumor immunity in glioma and hepatocellular carcinoma [28, 29]. CD161-overexpressing CD8⁺ T cells are enriched in chemoresistant breast cancer, and higher infiltration is linked to recurrence [30]. Docetaxel upregulates CLEC2D via the androgen receptor, reducing NK cell immunotherapy efficacy in castration-resistant prostate cancer [31]. Thus, CLEC2D may promote breast cancer chemoresistance by inhibiting tumor-infiltrating immune cells.

Notably, CLEC2D has been associated with better clinical outcomes in some cancer types. Sanchez-Canteli et al. demonstrated that although CLEC2D immunoreactivity in carcinoma cells correlated with poorer prognosis in HPV-negative oropharyngeal squamous cell carcinomas, the prognostic impact of CLEC2D mRNA levels from The Cancer Genome Atlas (TCGA) varied by HPV status and cancer type [12]. Similarly, TCGA analysis showed that CLEC2D expression was linked to improved prognosis in cervical squamous cell carcinoma [32]. In non-small cell lung cancer, CLEC2D is restricted to germinal center B cells in tertiary lymphoid structures, interacting with CD161⁺ CD4⁺ T cells to contribute to better outcomes [33]. The role of CLEC2D in the tumor microenvironment likely depends on the cell types expressing CLEC2D and the presence of CD161-positive immune cells, accounting for its context-dependent prognostic significance.

On the other hand, in the present study, CLEC2D immunoreactivity was significantly correlated with pT and Ki67 LI, and PR negativity. These findings suggest that CLEC2D is highly expressed in breast cancer with an aggressive phenotype characterized by higher invasion/proliferation activity as well as PR negativity (in ER-positive breast cancer). Consistently, CLEC2D correlates with WHO grade in glioma [8] and tumor size in renal cell carcinoma [13]. Based on TCGA data from 33 cancer types, including breast cancer, Zhu et al. reported that the CLEC2D/KLRB1 ratio is higher in most cancers and increases with tumor stage [34]. Additionally, in the present study, cell proliferation and migration activity of breast cancer cell lines were significantly suppressed by CLEC2D knockdown. Similarly, it has been reported that CLEC2D knockdown suppresses the proliferation of ccRCC cells [13]. Taken together, CLEC2D might not only suppress the cytotoxic activity of immune cells but also directly promotes the proliferation and migration of breast cancer cells, thereby promoting breast cancer progression and recurrence. In vitro assays showed that CLEC2D had a stronger effect on proliferation than migration, which is consistent with immunohistochemical findings showing a positive correlation with Ki67 but no association with lymph node metastasis. These findings suggest that CLEC2D plays a significant role, especially in cell proliferation. However, in the present study, we could not clarify the mechanisms underlying CLEC2D-related cell proliferation and migration in breast cancer. CLEC2D has been reported to form heterodimers with TLR2 [19], and we previously demonstrated a correlation between TLR2 immunoreactivity and increased proliferation and shorter disease-free survival in breast cancer [35]. In addition, both CLEC2D and TLR2 expression were upregulated in epirubicin-resistant MCF-7 cells, suggesting that the interaction between CLEC2D and TLR2 may play a partial role in the chemotherapy resistance of MCF-7 cells. Moreover, this study showed that CLEC2D regulated TLR2 mRNA expression in MCF-7 cells, suggesting it may promote dimerization with TLR2 via its upregulation. CLEC2D is associated with the regulation of NF-κB signaling and cytokine expression, including TNF-α and interferon-γ [21, 22, 36]. In addition, TLR2 is an estrogen-responsive gene [37], and several studies have reported that NFκB can enhance the transcriptional activity of ER [38]. Therefore, CLEC2D may not only directly promote TLR2 expression through NF-κB activation and cytokine secretion, but also enhance ER-mediated TLR2 expression via the same pathway.

In the present study, CLEC2D promoted both migratory and invasive abilities of MDA-MB-231 cells, whereas in MCF-7 cells, it enhanced migration but suppressed invasion. The wound healing assay mainly measures collective migration, while the Boyden chamber assay assesses single-cell migration associated with reduced cell–cell adhesion. In MCF-7 cells, CLEC2D likely helps maintain adhesion, so its knockdown may disrupt this and increase single-cell invasion. In contrast, MDA-MB-231 cells, having undergone epithelial to mesenchymal transition [39], are likely less affected by CLEC2D knockdown regarding adhesion. Additionally, the Boyden chamber assay with Matrigel requires extracellular matrix (ECM) degradation. Thus, in MCF-7 cells, CLEC2D may suppress ECM degradation. In MCF-7, an ER-positive cell line, ER signaling promotes matrix metalloproteinase expression [40], and CLEC2D might inhibit these effects, although their relationship remains unclear.

In summary, CLEC2D was overexpressed in breast cancer tissues and correlated with increased risk of recurrence of the patients, especially those who received chemotherapy. This might arise from the fact that CLEC2D not only suppresses antitumor effect of tumor-infiltrating lymphocytes but directly promotes breast cancer cell proliferation and migration.

## Supplementary Information

Below is the link to the electronic supplementary material.

Supplementary file1 (PDF 161 KB)

## Data Availability

All data and materials in the article are available from the corresponding author upon reasonable request.

## Declarations

### Research involving human and/or animals participants

Statement of human rights: All procedures performed in studies involving human participants were in accordance with the ethical standards of the institutional committee (Tohoku University Graduate School of Medicine) and with the 1964 Helsinki declaration and its later amendments or comparable ethical standards. For this type of study formal consent is not required. This article does not contain any studies with animals performed by any of the authors.
