# Supplementary material for: C-type lectin-like domain family 2 (CLEC2D) promotes proliferation and migration of breast cancer and serves as a poor prognostic factor
Source: Breast Cancer. 2025 Sep 12;33(1):88–98. doi: 10.1007/s12282-025-01777-5 (PMC12789214; doi:10.1007/s12282-025-01777-5)
Supplement: Supplementary file 1 — Supplementary file1 (PDF 161 KB) [file 12282_2025_1777_MOESM1_ESM.pdf]

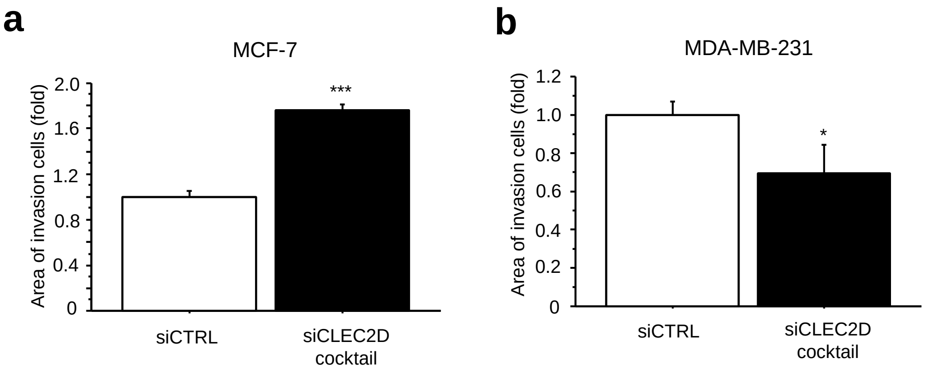

Supplementary Figure 1. Effects of CLEC2D knockdown on cell invasion in breast cancer cell lines. A Boyden chamber assay was performed using MCF-7 (a) and MDA-MB-231 (b) cells transfected with a CLEC2D-specific siRNA cocktail. 48 hours after transfection, cells were seeded into Matrigel-coated transwells and incubated for 48 hours (MCF-7) or 24 hours (MDA-MB-231). * $P < 0.05$, *** $P < 0.001$ vs. control (siCTRL). The data are presented as the mean ± S.D. ($n = 3$).

A Boyden chamber assay was performed using Corning® 6.5 mm Transwell® inserts with 8.0 µm pore polycarbonate membranes (Corning, NY, USA). MCF-7 and MDA-MB-231 cells, transfected with a CLEC2D-specific siRNA cocktail and suspended in serum-free medium 48 hours after transfection, were seeded into Matrigel (30 µg/well, Corning)-coated transwells. The lower chamber was filled with medium containing 10% FBS. The cells was incubated for 48 hours (MCF-7 cells) or 24 hours (MDA-MB-231 cells), after which the membranes were fixed with 10% neutral buffered formalin. Staining was performed using hematoxylin, and cells remaining on the inner surface of the transwell membrane—the side where cells were initially seeded—were gently removed using a cotton swab. The invasion cells were photographed using a microscope (100×, five fields per membrane), and the invasion cell area was quantified using ImageJ software (version 1.52a, https://imagej.nih.gov/ij/, accessed on 19 January 2025). The average invaded area was then calculated.

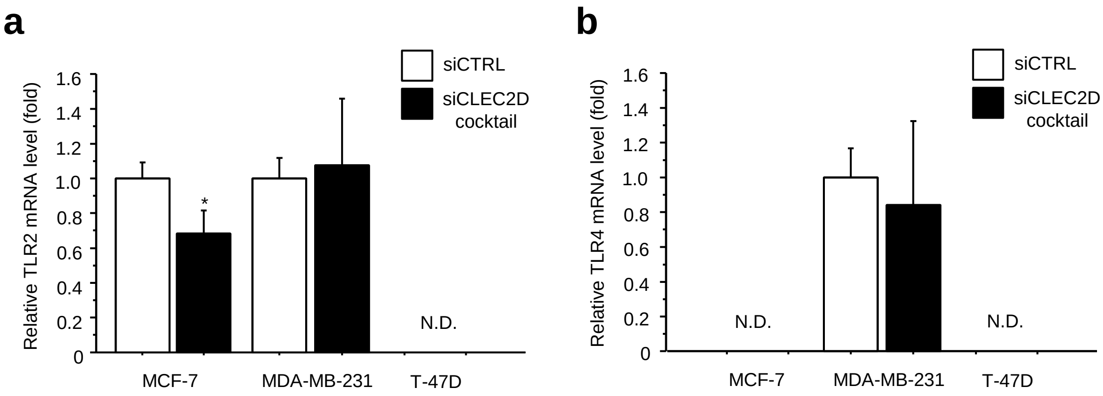

Supplementary Figure 2. The effects of CLEC2D knockdown on toll like receptor 2 (TLR2) and TLR4 in breast cancer cell lines. TLR2 (a) and TLR4 (b) mRNA levels in MCF-7, MDA-MB-231, and T-47D cells transfected with a CLEC2D-specific siRNA cocktail for 72 hours were evaluated by real-time PCR. * $P < 0.05$ vs. control (siCTRL). The data are presented as the mean ± S.D. ($n = 3$). mRNA levels were normalized to the expression of the housekeeping gene glyceraldehyde-3-phosphate dehydrogenase (GAPDH). N.D.; not detected (lower limit of detection).

Primer sequences are as follows. TLR2; forward 5’-AGGCGGACATCCTGAACCT-3’ and reverse 5’-GGCCAGCAAATTACCTGTGTG-3’, TLR4; forward 5’-ATAGCGAGCCACGCATTCA-3’ and reverse 5’-CATCCTCACTGCTTCTGTGAGC-3’.

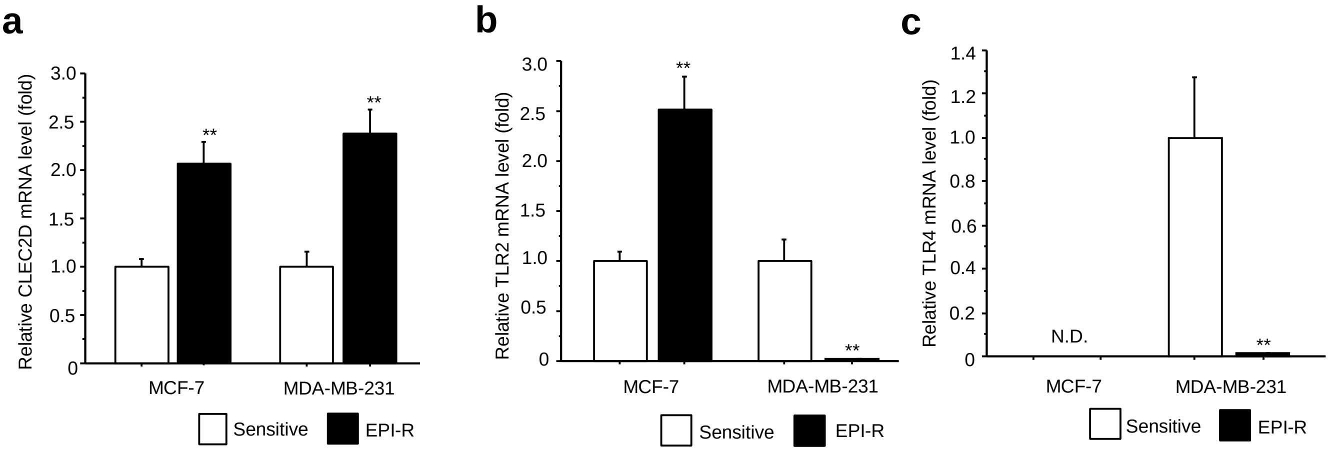

Supplementary Figure 3. The expression of CLEC2D (a), toll like receptor 2 (TLR2, b), and TLR4 (c) mRNA in epirubicin-resistant breast cancer cell lines. mRNA levels in epirubicin-resistant MCF-7 and MDA-MB-231 cells (EPI-R) were evaluated by real time PCR. * $P < 0.05$, $P < 0.01$ vs. their parental chemo-sensitive counterparts (Sensitive). The data are presented as the mean ± S.D. ($n = 3$). mRNA levels were normalized to the expression of the housekeeping gene glyceraldehyde-3-phosphate dehydrogenase (GAPDH). EPI-resistant breast cancer cell lines were established based on previous studies [1]. N.D.; not detected (lower limit of detection).

[1] Fujisawa S, Takagi K, Yamaguchi-Tanaka M, Sato A, Miki Y, Miyashita M, et al. Receptor for Hyaluronan Mediated Motility (RHAMM)/Hyaluronan Axis in Breast Cancer Chemoresistance. Cancers (Basel) 2024;16:3600.
